# Uptake and rollout of the World Health Organization-endorsed technologies for Tuberculosis diagnosis in Africa: a literature review of international evidence 2007-2021

**DOI:** 10.4314/ahs.v25i4.3

**Published:** 2025-12

**Authors:** Jean de Dieu Iragena, Esther Uwimaana, Derrick Semugenze, Kevin Komakech, Achilles Katamba, Anandi Martin, Moses Joloba, Willy Ssengooba

**Affiliations:** 1 Department of Immunology and Molecular Biology, Makerere University College of Health Sciences, School of Biomedical Sciences Kampala, Uganda; 2 Makerere University Lung Institute, Kampala, Uganda; 3 Department of Medical Microbiology, Makerere University College of Health Sciences Kampala, Uganda; 4 Department of Global Health and Amsterdam Institute for Global Health and Development, Amsterdam University Medical Centers Location University of Amsterdam, Amsterdam, The Netherlands; 5 Department of Medicine, Makerere University School of Medicine, Clinical Epidemiology and Biostatistics Unit; 6 Uganda Implementation Research Consortium, Kampala, Uganda; 7 Université Catholique de Louvain (UCLouvain), Institute of Experimental and Clinical Research, Brussels, Belgium; 8 Makerere University, Biomedical Research Center, Kampala, Uganda

**Keywords:** Tuberculosis (TB) diagnosis, Uptake and rollout, World Health Organization African Region (WHO/AFR), literature review

## Abstract

**Background:**

The World Health Organization (WHO) has endorsed a range of diagnostic tuberculosis (TB) over the years. A little is documented about the uptake in the WHO African Region (WHO/FR).

**Objective:**

We assessed the uptake of the endorsed diagnostic technologies for tuberculosis through a literature review.

**Methods:**

We reviewed literature in French and English from PubMed, Google Scholar, and Embase for TB diagnostics endorsed by WHO between January 2007 and December 2017, extending to December 2021 for recent technologies. We included publications from the 47 countries in the WHO/AFR. Data were analyzed using PRISMA diagrams and STATA 14.0.

**Results:**

Out of 3,399 articles, 1,716 articles were screened, and 92 qualified for analysis. The majority of articles were on Xpert MTB/RIF (XPERT) 22 (47%), Line Probe Assay (LPA), 10 (21%), and Mycobacteria Growth Indicator Tube (MGIT) 9 (19%). For rollout, 11 (24%) of countries had publications on Lipoarabinomannan (LAM) and 16 (36%) on XPERT. The median years for uptake were 6 for MGIT, 5 for XPERT, and 2.5 for LPA. For the rollout, the median years for MGIT, LPA, and XPERT were 7, 6, and 5 respectively

**Conclusion:**

Our study shows that the uptake and rollout are slow. Future studies should identify factors affecting rapid uptake and rollout.

## Introduction

In 2023, almost 2.5 million people fell ill with tuberculosis (TB) in the World Health Organization (WHO) African region; of those, almost 1.9 million new and relapse cases were notified (76%), leaving a gap of 24% undiagnosed, unnotified^1^. To address the End TB Strategy, the WHO has endorsed different diagnostic technologies over the years and defined the level of implementation to facilitate their uptake in tiers of laboratory systems^2^. The uptake varies across countries and depends on the complexity of the technology and minimum biosafety measures that should be implemented to reduce the risk of a laboratory-acquired infection^3^. Liquid culture systems, rapid speciation tests, and phenotypic Drug Susceptibility Testing of Second-Line Antituberculosis Drugs (pDSTSLDs) were endorsed in 2007, and 2008, respectively, but with limited use at National TB Reference Laboratories where biosafety was a requirement^4^. Along the same line, in 2008 and 2016, WHO respectively endorsed the use of line probe assay (LPA), the GenoTypeMTBDRplus for the rapid detection of Mycobacterium Tuberculosis Complex (MTBC) and simultaneous resistance to rifampicin (Rif) and isoniazid (H) for initial testing instead of phenotypic DST^5^-^6^ and GenoTypeMTBDRsl to detect resistance to Fluoroquinolones (FQs) and amikacin (Am) in patients with Rif-resistant/MDR-TB in less than 24 hours and to guide initiation of an appropriate MDR-TB treatment regimen^7^. In December 2010, WHO recommended the use of Xpert®MTB/RIF assay (referred to as Xpert, Cepheid, Sunnyvale, CA, USA) to represent a paradigm shift in the diagnosis of M. tuberculosis as well as rifampicin resistance-conferring mutations in about 2 hours, directly from sputum in adults presumptive of MDR or HIV-associated TB and was initially recommended for use at district and subdistrict laboratories^8^.

In 2011, light-emitting diodes (LED) microscopy was endorsed by WHO, which is 10% more sensitive and faster than conventional light microscopy (LM) using Ziehl-Neelsen staining^9^-^10^. Besides all, the magnitude of the HIV pandemic, the growing burden, and the spread of MDR-TB made microscopy irrelevant highlighting the need for investing in the early detection of TB and rifampicin resistance^11^.

In the same year 2011, a policy on Noncommercial culture and DST methods, such as microscopic observation of drug susceptibility (MODS), colorimetric redox indicator (CRI), and nitrate reductase assay (NRA) for screening patients at risk for MDR-TB was endorsed^12^.

In 2013, a new policy on Xpert assay with the addition of extrapulmonary TB was issued, expanding its use on all people suspected of TB, especially from nasogastric aspirate specimens in the pediatric population as an alternative to sputum^13^-^14^. Later in 2017, Xpert®MTB/RIF Ultra (Ultra) was developed and recommended for use to overcome the issue of the suboptimal sensitivity of Xpert in smear-negative sputum samples^15^.

In 2015, a policy on Lateral Flow Lipoarabinomannan (LAM) rapid diagnostic test (Alere Determine™ TB LAM Ag Alere Inc, Waltham, MA, USA) as a point-of-care test for the diagnosis and screening of active TB in the urine of people living with HIV has been issued^16^. Despite these recommendations and the evidence that implementation of AlereLAM reduces tuberculosis-related mortality, AlereLAM uptake has been slow due to its low sensitivity and specificity, restrictive eligibility criteria, reliance on CD4+ testing, and lack of advocacy and awareness^17^-^18^.

In 2016, a policy guidance was issued for TB LAMP [the Loopamp™ Mycobacterium tuberculosis complex (MTBC) detection kit, Eiken Chemical Company] for use as a rapid alternative to sputum-smear microscopy^16^.

In this literature review, we document the uptake, roll-out, and scale-up status of the WHO-endorsed technologies for TB diagnosis in Africa. We present the frequency of countries reporting uptake and roll-out and the number of years taken to publish on the uptake and roll-out of TB diagnostics after WHO endorsement.

## Methods

### Eligibility Criteria

#### Inclusion criteria

We included publications on implementation at central, regional, and peripheral levels. Studies in research labs, routine-based labs, communities, and research studies on the uptake and utilization of TB tests at the country level for which results were used for patient care were considered as roll-out (decentralization).

#### Exclusion criteria

We excluded articles for studies conducted outside the 47 countries in the WHO/AFR, those without a clear African setting or specific WHO-endorsed TB test name, non-TB-specific studies, studies not referring to uptake, rollout, and implementation, studies with TB tests endorsed before 2007 or after 2017 and implemented before 2007 or after 2021. We also excluded irrelevant studies, duplicates, studies with no full text, study protocols, case reports, poster presentations, studies missing years and levels of implementation, and studies with tests not yet endorsed by WHO. We excluded almost 50% as duplicates from the database search, and then 60% after screening by title and abstract for reasons mentioned above, and finally we excluded 50% of papers after full-text reading before reaching eligible records we considered for the analysis.

### Literature review registration and reporting

The review protocol was registered in the ISRCTN database (Reg. no.: ISRCTN24711056) and designed following the PRISMA 2020 Checklist^19^

### Search strategy

With articles published in French or English, through an electronic and manual search, we systematically reviewed the peer-reviewed literature on TB diagnostics (in humans) using PubMed, Google Scholar, and Embase. The diagnostics must have been endorsed by WHO between January 2007 and December 2017 and published until December 2021 in the 47 countries in the WHO African region^20^. For each diagnostic technology, we searched published articles per country with a focus on the uptake, utilization, roll-out, implementation, adoption, and operation with a combination of the diagnostic search terms as follows: Xpert®MTB/RIF, GeneXpert, GeneXpert MTB/RIF, Xpert, Ultra, NAAT, nucleic acid amplification test, CBNAAT, Cartridge Based nucleic acid amplification test; LAM, Urine LAM, LF-LAM, TB-LAM, Lateral Flow LAM, Urine antigen, Lateral flow urine lipoarabinomannan, FujiLAM, AlereLAM; LAMP, TB LAMP, TB-LAMP, Eiken, loop-mediated isothermal amplification, Loopamp MTBC; LED, Light-emitting diode fluorescent microscopy, auramine staining, LED-fluorescence microscopy, FluoLED; LPA, MTBDRplus, Genotype MTBDRplus, Genotype MTBDRsl Genotyping drug susceptibility testing, Hain, line probe assay, rpoB and katG genes, Hain Lifescience, Nipro; MGIT, BACTEC MGIT 960, BACTEC 960, Mycobacterium Growth Indicator Tube, liquid culture; MODS, Microscopic Observation Drug Susceptibility, nitrate reductase assay, NRA, colorimetric redox indicator, and CRI.

All retrieved articles were downloaded from the search database and uploaded into Rayyan systematic review software^21^. We used the “blind on” mode of the Rayyan software for screening. Four reviewers (JI, EU, KK, and DS) independently screened titles, abstracts, and full text in the “blind on” mode using predefined inclusion and exclusion criteria. A disagreement between reviewers was resolved through discussion by taking into account the level of scoring (included versus excluded) between reviewers to determine the final articles eligible for full-text reading. In the case of a persistent disagreement, a fifth reviewer (WS) was consulted as a tie-breaker to reach a consensus. Eligible articles were agreed upon by all reviewers and included in the analysis. At each screening stage, the number of excluded articles was recorded together with the reasons. All reviewers regularly met to determine if their approach was consistent with the objective. The PRISMA 2020 checklist (Table S1.) was used to document the study selection process

**Figure 1:**
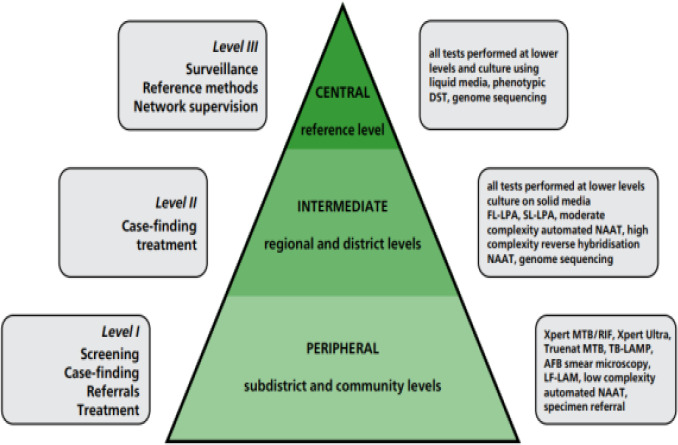
Organization of a TB diagnostic network^22^ Key: AFB: acid-fast bacilli; DST: drug susceptibility testing; FL: first-line; LAMP: loop-mediated isothermal amplification; LF-LAM: lateral flow lipoarabinomannan assay; LPA: line-probe assay; NAAT: nucleic acid amplification test; SL: second-line; TB: tuberculosis

The uptake refers to the action of taking up or making use of TB diagnostic technologies upon their availabilities following the endorsement and recommendations for use by the WHO and this showed articles talking about tests that were implemented at the central level versus those implemented at the intermediate (or regional) and peripheral levels of the TB laboratory network. We defined the roll-out of the test as it is used in the settings it was endorsed for by WHO. i.e. central, intermediate, or peripheral (Figure 1).

### Data extraction, synthesis and Analysis

We used a standard data extraction format prepared in Microsoft Excel (Microsoft Corp, Redmond, WA, USA). Data were extracted and described based on each article's details, including country name, a weblink per article, year of publication, title, test name, year of implementation, screening by (title, abstract, full article), and level of implementation. We specified if the implementation occurred at central, regional, and peripheral levels including the description of the setting according to the TB diagnostic network.

We estimated the frequency and median time for diagnostic uptake and roll-out by calculating the percentage of published papers per test per country out of the 47 countries in the WHO/AFR and the time (in years) taken for the uptake and rollout of a test respectively expressed as median years (Interquartile Range: IQR) calculated in taking into account the year of endorsement and uptake and/or rollout of a test. STATA 14 statistical software was used.

## Results

A total of 3,399 articles were obtained from the search databases. After the elimination of 1,683 duplicates, a total of 1,716 articles (S1 File) were recorded and screened (S1 File, S2). Of 1716 articles, 1034 were excluded to remain with 682 papers for full-text screening (S1 File, S3). We identified in total 343 articles (S1 File, S4) from which 92 articles (S1 File, S5) including 11, 14, 9, 3, 4, 31, 13, 4, and 2 tests were retained and eligible (at least one paper per country per technology) for the literature review analysis for MGIT, LPA, LED, MODS, NRA, XPERT, LAM, LAMP, and ULTRA tests respectively (Table 1).

**Table 1:** PRISMA flow diagram

Records Identified through database searching, total=3399 Duplicate Records Excluded, total= 1683 (50%) Records after duplicates removed, total=1716														
					Non-commercial culture and DST Methods									
Test name		MGIT	LPA	LED	MODS	NRA	CRI	XPERT	LAM	LAMP	ULTRA	Other tests	Unspecified	Total
Data base	PubMed	49	73	38	10	4	1	186	72	11	36	124	296	900
	Embase	10	32	7	6	1	0	61	87	2	3	9	109	327
	Others (google scholar, …)	49	78	62	3	2	0	220	13	5	1	16	40	489
Records Screened (Title & Abstract)	Total	**108**	**183**	**107**	**19**	**7**	**1**	**467**	**172**	**18**	**40**	**149**	**445**	**1716**
Reason for exclusion	Studies conducted outside of 47 countries in the WHO/AFR Studies without a clear setting in Africa (e.g.: absence of a country name) or a specific WHO-endorsed TB test name Non-TB-specific studies, studies missing years, and levels of implementation (central, intermediate, and peripheral) Studies that are not referring to the uptake, rollout, and im plementation; studies whose test is not yet endorsed by WHO Studies whose TB test was endorsed before 2007, and/or after 2017 and implemented before 2007, and/or after 2021 Studies that are irrelevant to TB diagnostic uptake (e.g.: treatment, Drug Resistance Surveys, etc.) Studies that do not show adoption and utilization in clinical practice (patient care purpose) or public health setting Irrelevant, duplicates, or no full text available Study protocol, case reports, conferences, meetings, and thesis													**1034 (60%)**
Reason for exclusion	Total	28	57	29	8	1	1	137	32	6	7	16	17	**339 (50%)**
	Outside of Africa, or no African country name	1	7	0	0	0	0	3	1	0	0	0	1	13 (4%)
	Reports, poster presentations and study protocols	1	2	1	0	0	0	3	3	0	0	0		10 (3%)
	Full text is not available	3	5	5	4		1	33	11	2	0	0	12	76 (22%)
	Irrelevant to TB diagnostic uptake	4	4	5	2	0	0	29	3	1	1	4	2	55(16%)
	Not used for patient care purpose	11	36	10	2	0	0	50	7	2	2	5	1	126 (37%)
	Year and level of implementation are missed	3	2	5		1	0	18	6	1	3	1	1	41 (12%)
	Test not yet recommended by WHO, or implemented before 2007, or endorsed after 2017	5	1	3	0	0	0	1	1		1	6	0	18 (5%)
Records included	Total	**24**	**42**	**32**	**4**	**4**	**0**	**153**	**60**	**6**	**10**	**1**	**7**	**343**
Eligible records for analysis	Total	**11**	**14**	**10**	**3**	**4**	**0**	**31**	**13**	**4**	**2**	**0**	**0**	**92**

Key: MGIT: Mycobacteria Growth Indicator Tube; LPA: Line Probe Assay; LED: Light Emitting Diode; MODS: Microscopic Observation Drug Susceptibility; NRA: Nitrate Reductase Assay; XPERT: Xpert MTB/RIF Assay; LAM: Lipoarabinomannan, LAMP: Loop-Mediated Isothermal Amplification, and ULTRA: Xpert MTB/RIF Ultra Assay

### Frequency of publications reporting the uptake and rollout of TB diagnostic tests

For the uptake, out of 47 countries, the majority of articles were for XPERT test 22 (47%), followed by LPA 10 (21%), and MGIT 9 (19%). For the rollout, 11 (24%) countries and 16 (36%) had publications on LAM and XPERT test use respectively (Table 2).

**Table 2:** Frequency of publications reporting uptake and rollout of TB diagnostic tests

Test name	Uptake n/N (%) (N=47)	Rollout n/N (%) (N=47)
**MGIT**	9 (19)	4 (9)
**LPA**	10 (21)	7 (16)
**LED**	7 (15)	7 (16)
**MODS**	2 (4)	1 (2)
**NRA**	2 (4)	2 (4)
**XPERT**	22 (47)	16 (36)
**LAM**	6 (13)	11 (24)
**LAMP**	3 (6)	2 (4)
**ULTRA**	1 (2)	2 (4)

Key: MGIT: Mycobacteria Growth Indicator Tube; LPA: Line Probe Assay; LED: Light Emitting Diode; MODS: Microscopic Observation Drug Susceptibility; NRA: Nitrate Reductase Assay; XPERT: Xpert MTB/RIF Assay; LAM: Lipoarabinmannan, LAMP: Loop-Mediated Isothermal Amplification, and ULTRA: Xpert MTB/RIF Ultra Assay.

### Time taken by a country to report uptake and rollout of TB diagnostic tests

The median years (Interquartile Range: IQR) for uptake were 6 (3.2 - 7.9), 5 (2.5 - 6.5), and 2.5 (3.2 - 7.9) for MGIT, XPERT, and LPA respectively, and lower for other tests. For the rollout, the median years for MGIT, LPA, and XPERT were 7 (3.0 – 7.0), 6 (1.6 - 9.4), and 5 (4.0 - 7.0) respectively, and lower for other tests (Table 3). The review was rigid about addressing publication bias, and the only way to minimize it was through consensus among the reviewers

**Table 3:** Time taken for uptake and rollout of TB diagnostic tests since endorsement by WHO

Test name	Year of endorsement	Median years (IQR) for Uptake	Median years (IQR) for Rollout
**MGIT**	2007	6 (3.2 - 7.9)	7 (3 - 7*)
**LPA**	2008	2.5 (1.3 - 8)	6 (1.6 - 9.4)
**LED**	2010	1.5 (1 - 7*)	2 (0 - 6*)
**MODS**		0.5 (0 - 1*)	2 (2 - 2*)
**NRA**		6 (5 - 7*)	1.5 (1 - 2*)
**XPERT**	2010	5 (2.5 - 6.5)	5 (4 - 7*)
**LAM**	2015	1.0 (0- 3*)	2 (0 - 4.4)
**LAMP**	2016	-	-
**ULTRA**	2017	-	2 (2 - 2*)

Key: MGIT:

Mycobacteria Growth Indicator Tube; LPA: Line Probe Assay; LED: Light Emitting Diode; MODS: Microscopic Observation Drug Susceptibility; NRA: Nitrate Reductase Assay; XPERT: Xpert MTB/RIF Assay; LAM: Lipoarabinomannan, LAMP: Loop-Mediated Isothermal Amplification, and ULTRA: Xpert MTB/RIF Ultra Assay.

* Lower (upper) confidence limit held at minimum (maximum) of sample

The number of publications on LAMP and XPERT uptake and rollout was small to get data represented hence the median box is empty

## Discussion

Our review showed that the uptake and rollout of TB diagnostic technologies upon the endorsement by WHO is slow, influenced by multiple factors in Africa. The review highlighted numerous publications on the uptake and rollout of MGIT, LPA, LED, XPERT, LAM, and LAMP as they become available.

XPERT had the highest uptake and rollout across Africa, being used at all levels of the laboratory network. Our results align with a study on the impact and cost of scaling up GeneXpert MTB/RIF in South Africa, which found a significant increase in TB cases diagnosed and treated after a wide implementation of Xpert MTB/RIF, demonstrating its high impact^23^. However, its uptake and rollout were delayed due to its initial limitation to high-risk groups of MDR and HIV-associated TB when it was recommended by WHO in 2010, before extending to extrapulmonary and children in 2017. Additionally, healthcare workers were slow to accept it as the first rapid molecular TB diagnostic test.

While MGIT and LPA were primarily implemented at central and regional levels in some countries due to biosafety requirements^24^, with long median times for uptake and rollout^25^-^26^.

For instance, LPA had a quick uptake (2.5 years) but a longer rollout (6 years) due to the need for specialized expertise, lengthy hands-on time, significant laboratory infrastructure, and not automated interpretation^27^. Laboratory operational delays were noted, including dependence on smear and culture positivity before MTBDRplus performance^28^.

LAM showed significant rollout, likely motivated by HIV programme support and its easy use as a point-of-care diagnostic test without additional biosafety requirements^17^.

Publications on the uptake and rollout of non-commercial culture and DST methods, including MODS and NRA, were few, likely due to their technical complexity, cost, and sophisticated laboratory infrastructure requirements^12^.

LED microscopy had a shorter median implementation time at all levels, requiring no extra infrastructural and minimal training, as personnel already had microscopy skills. LED's diagnostic gain over conventional light microscopy among HIV-positive likely motivated its rapid uptake and roll-out^29^.

Although the LAMP test has good sensitivity and specificity, it is generally lower than that of the XPERT assay^30^ and does not provide a drug resistance profile, leading to slow uptake and limited publications.

ULTRA had a quicker rollout (within 2 years) as the GeneXpert system was already available and familiar to healthcare workers.

The slow uptake and rollout of the WHO-endorsed TB diagnostics in Africa highlight the need for improved infrastructure and training including, learning from successful models like XPERT. Countries, with remarkable uptake and rollout regularly participated in TB diagnostic validation studies before WHO endorsement, facilitating rapid acceptability and roll-out. Francophone countries where most publishing journals are in English, may have faced publication bias, limiting the opportunity to publish articles on uptake and roll-out.

Barriers and challenges linked to technical complexities, infrastructure requirements, funding and donor dependency, healthcare worker training, and acceptance must be addressed to speed up the process of uptake and rollout.

Enhancing technical assistance, capacity building, infrastructure development, and tailored strategies to address specific barriers can mitigate slow uptake and rollout. Influencing research settings for validation and WHO Prequalification studies can raise early awarenees of technologies. Economic situations, political commitments, and instabilities may also impact early validation studies, uptake, and roll-out.

Our study focuses on WHO diagnostic technologies endorsed between 2007 and 2017. Publications up to 2021 were allo included, with other technologies endorsed between 2017 and 2021 influencing some countries'development and readiness for uptake and rollout.

In 2019, new evidence justified the use of a new urine-based test, Fujifilm SILVAMP TB LAM, with 30% higher sensitivity than AlereLAM, leading WHO to issue an updated policy^31^.

In 2020, Molbio Diagnostics, developed the TrueNat molecular TB diagnostic tool for detecting TB and RIF-resistance within an hour^32^.

WHO'2020 systematic review recommended three classes of technology based on implementation complexity and target conditions^33^.

In 2021, Cepheid improved the GeneXpert instrument's multiplexing capacity, detecting more molecular targets with the Xpert MTB/XDR test, requiring the latest instrument with 10-color optics^34^.

Next-generation sequencing (NGS) and bioinformatics pipelines have been developed for rapid detection of M. tuberculosis drug resistance, with sequence data aiding public health through understanding transmission patterns^35^.

Post-2017 endorsed technologies contributed to TB lab strengthening in Africa, with specific implementation requirements. Rapid technological advances brought new and better technologies, changing WHO TB diagnostic policies and potentially distracting from previously endorsed technologies. However, these advances offer hope for achieving the End TB strategy milestones and targets. Our stusdy's strength lies in the review team's extensive experience in TB diagnostic-related research, conducting literature reviews, and summarizing diverse literature. The study covered the 47 countries in the WHO AFR making findings more generalizable.

Limitations include donor-driven uptake of diagnostic technologies, delayed or non-existent implementation or publication, and potential publication bias due to language. Some low-burden countries were excluded, and a review combining articles with key informant or survey interviews could provide stronger findings.

## Conclusion

This literature review highlights the varied uptake and rollout of WHO-endorsed TB diagnostic technologies across the African region. While some technologies like Xpert MTB/RIF and LED microscopy have seen significant implementation, others face challenges due to technical complexity and infrastructure requirements. The findings underscore the need for enhanced technical assistance to capacitate countries in publishing their findings, funding, and tailored strategies to address the unique barriers faced by different countries. Future efforts should focus on comprehensive surveys to reach out to people at a country level and studies to better understand and mitigate factors influencing the uptake and rollout of TB diagnostic tools. Furthermore, designing a quick guide for technology uptake and roll-out after WHO endorsement may offer support towards technical know-how for rapid uptake and roll-out among high TB endemic countries. This may also inform the uptake and roll-out of other disease diagnostic technologies.
